# Dietary supplement usage and motivation in Brazilian road runners

**DOI:** 10.1186/s12970-014-0041-z

**Published:** 2014-08-21

**Authors:** José Vítor Vieira Salgado, Pablo Christiano Barboza Lollo, Jaime Amaya-Farfan, Mara PatríciaTraina Chacon-Mikahil

**Affiliations:** 1 Exercise Physiology Laboratory - FISEX, FEF-UNICAMP Cidade Universitária, Physical Education Faculty, State University of Campinas, Cep:13083-851, Campinas SP 6134, Brazil; 2 Food’s Engineering Faculty, Department of Food and Nutrition, State University of Campinas - UNICAMP, Campinas, SP, Brazil; 3 Sport’s Sciences Department, University of Campinas, Physical Education Faculty, Erico Veríssimo Av., 701., Campinas, Brazil

**Keywords:** Road race, Road runners, Dietary supplements, Performance, Running

## Abstract

**Background:**

The consumption of dietary supplements is highest among athletes and it can represent potential a health risk for consumers.

**Objective:**

The aim of this study was to determine the prevalence of consumption of dietary supplements by road runners.

**Methods:**

We interviewed 817 volunteers from four road races in the Brazilian running calendar. The sample consisted of 671 male and 146 female runners with a mean age of 37.9 ± 12.4 years.

**Results:**

Of the sample, 28.33% reported having used some type of dietary supplement. The main motivation for this consumption is to increase in stamina and improve performance. The probability of consuming dietary supplements increased 4.67 times when the runners were guided by coaches. The consumption of supplements was strongly correlated (r = 0.97) with weekly running distance, and also highly correlated (r = 0.86) with the number of years the sport had been practiced. The longer the runner had practiced the sport, the higher the training volume and the greater the intake of supplements. The five most frequently cited reasons for consumption were: energy enhancement (29.5%), performance improvement (17.1%), increased level of endurance (10.3%), nutrient replacement (11.1%), and avoidance of fatigue (10.3%). About 30% of the consumers declared more than one reason for taking dietary supplements. The most consumed supplements were: carbohydrates (52.17%), vitamins (28.70%), and proteins (13.48%).

**Conclusions:**

Supplement consumption by road runners in Brazil appeared to be guided by the energy boosting properties of the supplement, the influence of coaches, and the experience of the user. The amount of supplement intake seemed to be lower among road runners than for athletes of other sports. We recommend that coaches and nutritionists emphasise that a balanced diet can meet the needs of physically active people.

## Background

The consumption of dietary supplements is highest among athletes [1],[2]. The practice has become increasingly popular in Brazil, and is prevalent not only among athletes but among those who practice physical activity for recreational purposes and non-professional athletes [3] such as road runners [4]. The abusive consumption of food supplements can represent a health risk for consumers in general and also for road runners. The analysis of dietary supplements conducted by the International Olympic Committee’s anti-doping lab found that of 634 supplements tested, 14.8% contained precursors of hormones such as testosterone and nandrolone, substances not declared on the product labels. Similar findings have been reported by other authors [5].

Both the supply of dietary supplements and road racing are growing trends, as can be seen from the number of competitions and the steady increase in the number of participants [6],[7] since the “jogging boom” of the early 1970s, as inspired by the theory of Kenneth Cooper [8]. According to the Marathons and Distance Races International Association [9], both marathons and road races are increasingly being seen as participative recreations. This is evident in Brazil, especially in São Paulo City where road races have grown exponentially; in 2012 there were 311 competitions compared with 11 in 2001 [5],[6]. Thus, the objective of this study was to verify the prevalence of the use of commercial dietary supplements among Brazilian road runners.

## Methods

A previously structured interview was conducted with 817 registered runners who agreed to participate voluntarily in the study and signed a consent term. The research project was previously approved by the UNICAMP university’s Ethics Research Committee (n° 5372005). The targeted group of runners took part in the following races in the national official calendar of competitions (Table 1): “Integração” race (10 km), Campinas-SP; “Maratona Pão de Açúcar de Revezamento” race (10 km), São Paulo-SP; “Volta Internacional da Pampulha” race (17,8 km), Belo Horizonte-MG; and “São Silvestre” race (15 km), São Paulo-SP. The average age (±SD) of the sample was 37.9 ± 12.4 years. The oldest subject interviewed was 92 and the youngest was 15 years old. Of all the subjects interviewed, 82.1% (n = 671) were men and 17.9% (n = 146) were women.

**Table 1:** Road races where the data were collected

**City**	**State**	**Road race**	**Distance**
Campinas	SP	Integração	10 km
São Paulo	SP	Maratona Pão de Açúcar de Revezamento	42,195 km
Belo Horizonte	MG	Volta da Pampulha	17,8 km
São Paulo	SP	São Silvestre	15 km

### Sample selection

The sample design used was defined according to the calculation of the sample size to a proportion, considering a value of 50% for the proportion (p = 50%) for the athletes to submit to nutritional supplementation, with a variation of 7% (v = 7%) and confidence level of 5% (alpha = 5%), also considering 10% of loss and rejection. Thus a total of 840 interviews was determined as necessary, distributed throughout the four races (210 interviews/race). The sampling was random for each race, and only amateur athletes were considered for filling in the questionnaire.

### Questionnaire

The questionnaire used for the interviews was structured according to a pre-competition scenario, following the recommendations of Foddy, 1994 [10]. The questionnaire comprised 10 questions, including both closed questions such as, “Do you use any type of food supplement?” (with reply options of “yes” or “no”), and open questions such as, “Which supplement(s) do you use?” The questionnaire was tested and validated before its application, and the validation (pre-test) and training of the evaluation team took place at the “Corrida da Independência” in Campinas - SP, Brazil.

### Data analysis

A descriptive analysis, inferential statistics and hypothesis test: Pearson’s Correlation test and the Odds Ratio (OR) test were carried out using the software SPSS 13.0 for Windows, Release 13.0, and adopting a value of p < 0.05.

## Results

The average age (±SD) of the sample was 37.9 ± 12.4 years. The oldest interviewed subject was 92 and the youngest 15 years old. Of all the interviewed subjects, 82.1% (n = 671) were men and 17.9% (n = 146) were women.

Those who reported to be dietary supplement consumers (n = 230) represented 28.3% of the entire sample, of which 81.7% were men (n = 188) and 18.3% women (n = 42). Of the total sample, 71.7% (n = 687) were non-consumers of supplements, constituted by men 82.3% (n = 483) and 17.7% (n = 104) women. We did not find any significant differences between genders among dietary supplement consumers and non-consumers.

**Figure 1:**
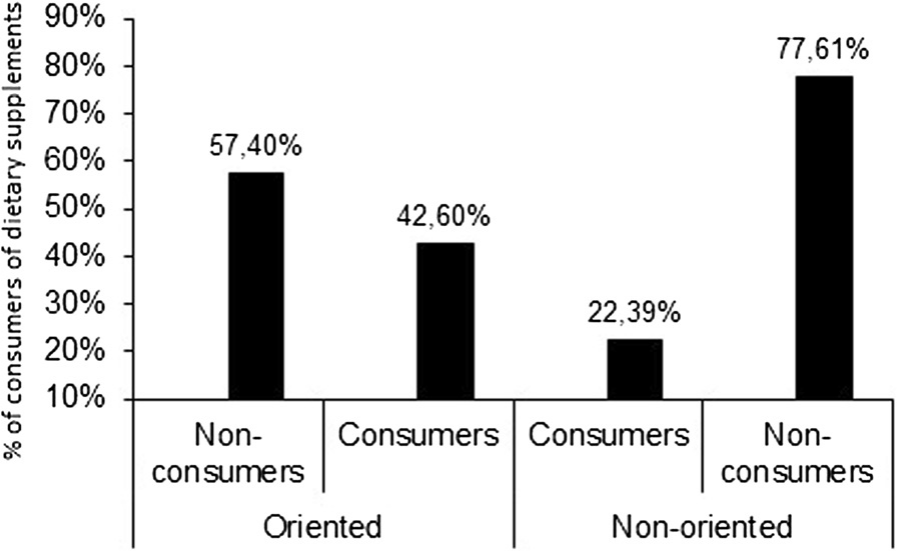
Percentages of dietary supplement consumers and non-consumers in relation to orientation (or not) by professionals with respect to the practice of physical activity of 817 Brazilian road runners.

When asked whether they received some guidance from managers or coaches in their physical activities, 27.3% of the subjects stated that they did not receive guidance from either source. Among those with professional guidance for race training, the dietary supplement consumers represented almost twice the number of those with no guidance (42.60 to 22.39%), as seen in Figure 1.

**Figure 2:**
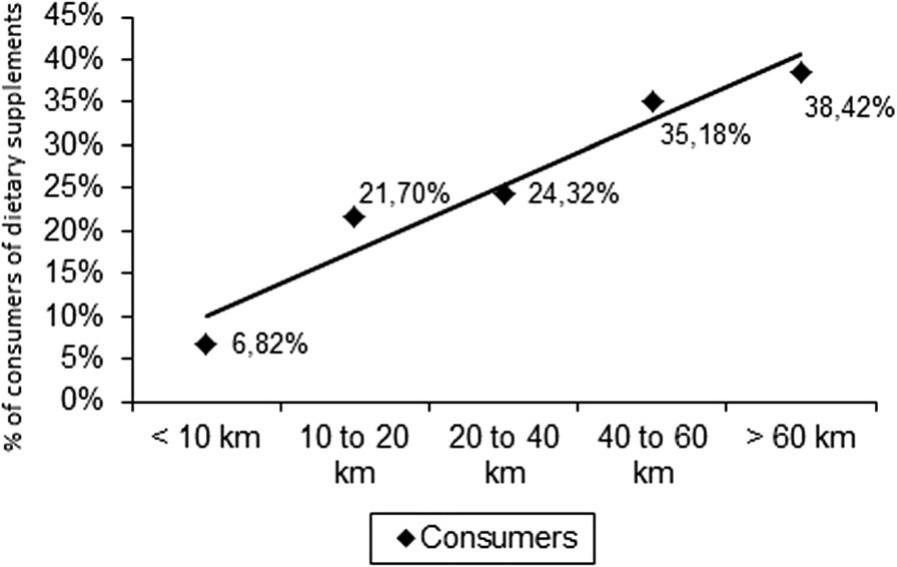
Pearson’s Correlation between dietary supplement consumption and training volume (km/week) in 817 Brazilian road runners.

The fact that a runner had been trained by a coach increased the chances of the runner being a dietary supplements consumer (OR = 4.67, p < 0.0001) by more than 4.5 times. When correlating the dietary supplements consumption with the volume of weekly training (km/week), the coefficient found was r = 0.97, indicating that the higher the training volume, the higher the frequency of dietary supplement consumption (Figure 2).

**Figure 3:**
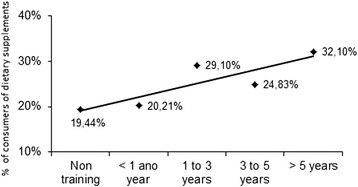
Pearson’s Correlation between dietary supplement consumption as a function of the road racer’s history of practice in 817 Brazilian road runners.

A similar tendency can be found in Figure 3, where the frequency of consumers was plotted against the number of years of road-racing practice. The correlation in this case was r = 0.86. Therefore, consumers of supplements were more frequently found among subjects that had been regularly training for a longer period of time, to the detriment of beginners.

As noticed in Figure 3, only about 20% of the runners with no specific training for road races, though frequently participating in competitions, did consume supplements. Comparing the prevalence of dietary supplement consumption separately in each race, significant differences were observed only among competitors of the São Silvestre and the Integração competitions (p = 0.003), as seen in Table 2.

**Table 2:** Supplements used by the athletes in 4 road races in Brazil

	**Integração**	**PA**	**Pampulha**	**SS**	**TOTAL**
Non consumers	79.6%	69.0%	73.0%	66.0%	81.7%
Consumers	20.4%	31.0%	27.0%	34.0%	28.3%
Vitamins and Minerals	16.7%	25.8%	29.8%	42.5%	28.7%
Carbohydrate	26.7%	16.1%	17.0%	10.0%	17.5%
Protein	20.0%	12.9%	17.0%	15.0%	16.2%
BCAA*	33.3%	29.0%	21.3%	20.0%	25.9%
Creatine	3.3%	12.9%	10.6%	10.0%	9.2%
Others	0.0%	3.2%	4.3%	2.5%	2.5%

*Branched chain amino acids.

Of the subjects using supplements (28.3% of the sample), the five reasons mentioned as causes for supplement consumption were: to obtain energy (29.5%; n = 69), to increase performance (17.1%; n = 40), to replace nutrients (11.1%; n = 26), to increase stamina (10.3%; n = 24), and to avoid fatigue (10.3%; n = 24). Of the consumers, 29.5% (n = 69) used more than one supplement and declared more than one reason for consuming them.

We have noticed that behind the reasons quoted to induce consumption (obtaining energy, increasing stamina, and avoiding fatigue), there were allusions to boosting performance. Grouping the reasons, the athletic performance was the most quoted one; i.e., 17.1% and 54.7% of the consumers, respectively, quoted it either straightforwardly or indirectly. The dietary supplements that the road runners admitted using the most were carbohydrates (52.17%).

From the reasons quoted (obtaining energy, increasing stamina, and avoiding fatigue), it was thought that the enhancement of performance was the main factor in taking supplements. Grouping the reasons, athletic performance was the most quoted, with 17.1% and 54.7% of consumers respectively quoting it either straightforwardly or indirectly. We found only five subjects who mentioned the consumption of isotonic drinks, but this low number may result from the non-association of isotonic drinks with dietary supplements.

## Discussion

The aim of the present study was to verify the prevalence of use of commercial dietary supplements amongst Brazilian road runners and 28.3% of the road runners questioned were found to be dietary supplement consumers. No studies of a similar population (Brazilian road runners) were found, but the percentage of road runners (amateurs) consuming dietary supplements in this study was similar to the prevalence found in the Los Angeles Marathon of 1987, which was 29% [11]. This prevalence is significantly low as compared to elite athletes, the use of nutritional supplements in professional long and middle distance track and field athletes being 82% [12]. The general use of nutritional supplements by athletics has been reported to be about 60% in adults and junior athletes [13]. This suggests that supplement consumption in Brazil is a relatively recent phenomenon, and lower than in developed countries. The data show there is a substantial consumption of dietary supplements amongst road runners, although lower than amongst gym goers (36.8%) [14]–[16] and basketball players (58%) [17]. Studies carried out with samples that were equally significant to those used in the present study [14],[15] reported consumption rates in gyms that varied between 36.8 and 61.2%. The greater consumption amongst runners with professional coaching was obvious, suggesting that physical education teachers or managers may be stimulating this consumption in some way. The authors would like to clarify that in this study the interviewees were not asked to reveal who recommended this practice. However, data from gyms clearly identified these professionals as strong inducers of supplement consumption [14]–[18]. For an adequate consumption, road racers should ask for professional advice in the use of supplements, since some supplements have been shown to contain doping substances [5], and it is unclear whether the coaches or athletes know about this.

We detected only five subjects who mentioned the consumption of isotonic drinks, but this low number may result from the non-association of isotonic drinks with dietary supplements. During races, these products are frequently handed out for free. To the contrary of the present data, the consumption of isotonic drinks was 32% amongst gymnasium users [14], 27.5% amongst university students [19] and 90.1% amongst university athletes from Singapore [20]. The present study showed that the frequency of consumption increased with either the distance of weekly training or the training time. This outcome may be related to the perception that this type of training is associated with high energy requirements.

Considering that the races analyzed covered a minimum distance of 10 km, hydration and electrolyte loss may be considered as relevant factors, since about 2% dehydration already causes an important loss of performance, dehydration between 4 and 6% may cause thermal fatigue, and dehydration above 6% causes a risk of thermal shock, coma and even death [21]. Nevertheless, amongst those interviewed, only 2.1% (n=5) reported straightforwardly having consumed some kind of supplement with this intention. The authors believe that in addition to the above, the higher exposure of newcomers to environments where supplement consumption is commonplace could place the more experienced athlete in a truly influential position to promote new dietary techniques, in order for beginners to achieve their desired performances. Supplement consumption by road runners in Brazil appears to have been guided by the energy boosting properties of the supplement (38.6%), the influence of physical educators, the training volume and by the experience of the user in road races. The volumes intakes seemed to be lower than those practiced by athletes of other sports.

## Conclusion

The authors believe that the higher exposure of newcomers to environments where supplement consumption is commonplace could place the more experienced athlete in an influential position to promote new dietary techniques, in order for beginners to achieve their desired performance. Supplement consumption by road runners in Brazil appears to have been guided by the energy boosting properties of the supplement, the influence of coaches, the training volume, and the experience of the user in road races. The amount of supplement intake seemed to be lower than for athletes of other sports. Coaches and nutritionists should emphasise that a balanced diet can meet the needs of physically active people to avoid inadequate use of dietary supplements by road racers.

## Competing interests

The authors declare that they have no competing of interest.

## Authors’ contributions

Conception and design of the study by JVVS and MPTCM. Generation, collection, assembly, analysis and interpretation of data by JSVV and PCBL. Revision of the manuscript by JAF. Approval of the final version of the manuscript by MPTCM. All authors read and approved the final manuscript.

## Acknowledgments

The authors are thankful to CNPq/SAE-UNICAMP and Physical Education Faculty of University of Campinas for the support, and all the runners that participated in this study, as well as the team involved in the data collection.

## References

[B1] Braun H Koehler K Geyer H Kleiner J Mester J Schanzer W Dietary supplement use among elite young German athletes Int J Sport Nutr Exerc Metab 2009 19 97 109 19403956 10.1123/ijsnem.19.1.97

[B2] http://www.ncbi.nlm.nih.gov/pubmed/24667342 Wiens K, Erdman KA, Stadnyk M, Parnell JA: **Dietary supplement usage, motivation, and education in young, Canadian athletes.** *Int J Sport Nutr Exerc Metab* 2014, . 10.1123/ijsnem.2013-0087 24667342

[B3] Dietary changes, fluid replacement, dietary supplements and drugs: demonstration of ergogenic action and potential health risks Rev Bras Med Esporte 2003 9 52 58

[B4] Salgado JVV Lollo PC Miyasaka CK Chacon-Mikahil MPT Prevalence of the Dietary Supplements Intake in Brazilian Road Runners13th annual congress of the European College Sport Science 2008 1 Taylor & Francis in Estoril, Portugal 718

[B5] Kohler M Thomas A Geyer H Petrou M Schanzer W Thevis M Confiscated black market products and nutritional supplements with non-approved ingredients analyzed in the Cologne Doping Control Laboratory 2009 Drug Test Anal 2010 2 533 537 10.1002/dta.186 21204286

[B6] Hespanhol Junior LC Costa LOP Carvalho ACA e Lopes AD A description of training characteristics and its association with previous musculoskeletal injuries in recreational runners: a cross-sectional study Rev Bras Fisioter [online] 2012 16 46 53 10.1590/S1413-35552012000100009 22441228

[B7] http://libdigi.unicamp.br/document/?code=000467613 Salgado JVV: **Comparison of functional and biochemical indicators in middle aged men undergoing aerobic training and long distance runners.** *Ms thesis. University of Campinas, Physical Education Faculty* 2009, .

[B8] Salgado JVV Chacon-Mikahil MPT Street race: analyses of the growth of the number of competitions and pratictioners Conexões 2006 4 100 109

[B9] AIMS 1982 – 2007: *Association of International Marathons and Distance Race: 25 Years of Running History.* 2007.

[B10] Foddy L Mantle J Constructing questions for interviews and questionnaires: theory and practice in social research 1993 Cambridge University Press, Cambridge

[B11] Nieman DC Gates JR Butler JV Pollett LM Dietrich SJ Lutz RD Supplementation patterns in marathon runners J Am Diet Assoc 1989 89 1615 1619 2809038

[B12] Tscholl P Alonso J Dolle G Junge A Dvorak J The use of drugs and nutritional supplements in top-level track and field athletes Am J Sports Med 2010 38 133 140 10.1177/0363546509344071 19812387

[B13] Corrigan B Kazlauskas R Medication use in athletes selected for doping control at the Sydney Olympics (2000) Clin J Sport Med 2003 13 33 40 10.1097/00042752-200301000-00007 12544162

[B14] Goston JL Correia MI Intake of nutritional supplements among people exercising in gyms and influencing factors Nutrition 2010 26 604 611 10.1016/j.nut.2009.06.021 20004078

[B15] Maughan RJ Depiesse F Geyer H The use of dietary supplements by athletes J Sports Sci 2007 25 Suppl 1 S103 S113 10.1080/02640410701607395 18049988

[B16] Lollo PCB Tavares MCGCF Profile of the consumers of dietary supplements in the fitness centers of Campinas Rev Dig 2004 5 105 111

[B17] Schroder H Navarro E Mora J Seco J Torregrosa JM Tramullas A The type, amount, frequency and timing of dietary supplement use by elite players in the First Spanish Basketball League J Sports Sci 2002 20 353 358 10.1080/026404102753576134 12003281

[B18] Smith-Rockwell M Nickols-Richardson SM Thye FW Nutrition knowledge, opinions, and practices of coaches and athletic trainers at a division university Int J Sport Nutr Exerc Metab 2001 11 174 185 11402251 10.1123/ijsnem.11.2.174

[B19] Malinauskas BM Overton RF Carraway VG Cash BC Supplements of interest for sport-related injury and sources of supplement information among college athletes Adv Med Sci 2007 52 50 54 18217389

[B20] Tian HH Ong WS Tan CL Nutritional supplement use among university athletes in Singapore Singapore Med J 2009 50 165 172 19296032

[B21] Shirreffs SM The importance of good hydration for work and exercise performance Nutr Rev 2005 63 S14 S21 10.1111/j.1753-4887.2005.tb00149.x 16028568

